# Running exercise with and without calcium supplementation from tuna bone reduced bone impairment caused by low calcium intake in young adult rats

**DOI:** 10.1038/s41598-023-36561-y

**Published:** 2023-06-13

**Authors:** Panan Suntornsaratoon, Thachakorn Thongklam, Thaweechai Saetae, Buapuengporn Kodmit, Sarawut Lapmanee, Suchinda Malaivijitnond, Narattaphol Charoenphandhu, Nateetip Krishnamra

**Affiliations:** 1 Department of Physiology, Faculty of Science, Mahidol University, Bangkok, Thailand; 2 Center of Calcium and Bone Research, Faculty of Science, Mahidol University, Bangkok, Thailand; 3 Global Innovation Center, Thai Union Group Public Company Limited, Bangkok, Thailand; 4 Department of Biology, Faculty of Science, Chulalongkorn University, Bangkok, Thailand; 5 Department of Basic Medical Sciences, Faculty of Medicine, Siam University, Bangkok, Thailand; 6 National Primate Research Center of Thailand, Chulalongkorn University, Saraburi, Thailand; 7 Institute of Molecular Biosciences, Mahidol University, Nakhon Pathom, Thailand; 8 The Academy of Science, The Royal Society of Thailand, Dusit, Bangkok, Thailand

**Keywords:** Physiology, Diseases, Endocrinology

## Abstract

Inadequate calcium intake during childhood and adolescence is detrimental to bone metabolism. Here, we postulated that calcium supplement prepared from tuna bone with tuna head oil should benefit for skeletal development than CaCO_3_. Forty female 4-week-old rats were divided into calcium-replete diet (0.55% w/w, *S1*, n = 8) and low-calcium groups (0.15% w/w for 2 weeks; *L*; *n* = 32). Then *L* were subdivided into 4 groups (8/group), i.e., remained on *L*, *L* + tuna bone (*S2*), *S2* + tuna head oil + 25(OH)D_3_ and *S2* + 25(OH)D_3_. Bone specimens were collected at week 9. We found that 2 weeks on low calcium diet led to low bone mineral density (BMD), reduced mineral content, and impaired mechanical properties in young growing rats. Intestinal fractional calcium absorption also increased, presumably resulting from higher plasma 1,25(OH)_2_D_3_ (1.712 ± 0.158 in *L* vs. 1.214 ± 0.105 nM in *S1*, *P* < 0.05). Four-week calcium supplementation from tuna bone further increased calcium absorption efficacy, which later returned to the basal level by week 9. Calcium supplementation successfully restored BMD, bone strength and microstructure. However, 25(OH)D_3_ + tuna head oil + tuna bone showed no additive effect. Voluntary running also effectively prevented bone defects. In conclusion, both tuna bone calcium supplementation and exercise are effective interventions for mitigating calcium-deficient bone loss.

## Introduction

Osteoporosis is one of the diseases that cause disability with serious life-threatening complications, especially in elderly people^1^. The prevalence of osteoporotic fracture appears to be increasing in both men and women in developing countries as a result of an increase in the aging population. Indeed, postmenopausal women exhibit greater risk of osteoporosis and fracture than males owing to a rapid decline of circulating estrogen, which normally exerts several anabolic actions on bone such as enhancement of osteoblast-mediated bone formation. However, calcium supplementation in the elderly does not produce much improvement in bone if started later in life^2^. Adequate calcium intake is particularly important in adolescence because this period is critical for acquiring bone mass^3^. Normally, the accrual of bone mass begins in childhood and continues until it reaches a peak between the age of 20 and 30 years^4^. Peak bone mass—defined as the amount of bone present at the end of the skeletal maturation—is one of the determinants of the onset of osteoporosis (review article^5^). However, once peak bone mass is attained, there after bone calcium content gradually decreases with age, particularly in women whose bone loss is rapid after menopause^6^. Therefore, an optimal amount of calcium in bone or high peak bone mass is important and could prevent individuals from developing osteoporosis later in life.

Since calcium is a mineral, it is obtained only from the diet through absorption. However, the intestinal absorption of calcium is not very efficient and is influenced by many factors. It is stimulated by vitamin D_3_, sugars, some amino acids and certain short-chain fatty acids^7–9^. Because calcium and vitamin D are vital for bone development, calcium and vitamin D deficiencies in children and adolescents (age 2–18 years; a period of fast growth) lead to lower peak bone mass and higher risk of bone fracture later in life^10^ and review article^11^. The leading causes of vitamin D deficiency in children and adolescents are less time spent outdoors, overuse of sunscreen protections and inadequate intake of food rich in vitamin D, particularly fish and functional foods (nutrients that provide health benefits beyond basic nutrition which can reduce the risk of noncommunicable diseases^12^. It was reported that higher intake of calcium was associated with significant gains in hip bone mineral density (BMD) in young female runners^13^, increases in bone mineral status in adolescent girls^14^, and bone mineral density in Gambian children who had been on low calcium diet^15^.

Our previous investigation showed that calcium in hydroxyapatite form had greater bioavailability than calcium carbonate. Moreover, calcium supplementation from tuna bone was able to prevent lactation-induced bone loss in experimental animals^16^. Thus, fish bone is considered a good natural source of calcium supplementation. Normally, the efficiency of the intestinal absorption of calcium is about 20% of total intake, and this efficiency increases upon vitamin D_3_ stimulation^17^. Besides, vitamin D_3_ is essential for the musculoskeletal development and bone formation. Since crude tuna head oil contains a high amount of vitamin D_3_ and Omega-3 polyunsaturated fatty acids^18^, the latter of which, have been known to improve cognitive function and increase neuroplasticity, tuna fish oil could be one of the most effective and readily available sources of vitamin D supplement. In addition, Omega-3, essential fatty acids, especially those from marine oils, could stimulate intestinal calcium absorption in vitro study as well as promote bone growth in young growing age^19,20^. Since females have greater risk of fragility fracture and osteoporosis than males, especially after menopause and becoming aging^5^, and since early bone health and peak bone mass in young adult potentially determine osteoporosis risk of females later in life^6^, we sought to develop calcium supplement products for females. In the present study, we therefore performed all experiments only in female animals.

It is well recognized that regular exercise provides several physiological and psychological benefits. Previous experiment showed that weight-bearing exercise, such as forced treadmill running, in ovariectomized rats helped prevent reduction in trabecular bone volume and bone mineral density^21^, and even increased bone volume and thickness, osteoblast activity, and improved dynamic bone formation^22^.

The conceptual idea of this study is to provide a new form of calcium supplement from a natural source for use during childhood and adolescence, i.e., tuna bone (calcium-rich product) and tuna head oil (vitamin D-rich product), and to evaluate the beneficial effect of impact exercise alone or combined with calcium-oil supplementation on bone microstructure and growth in young female growing rats. Combining exercise with calcium supplementation may provide additive effect on loaded bone, particularly tibiae and femora.

## Results

### Low dietary calcium intake for 2 weeks resulted in hypocalcemia with hyperphosphatemia and development of osteoporosis, that got worse if calcium insufficiency continued for another 11 weeks

In this section, there were 2 calcium diet formulae, i.e., low calcium diet (0.15% Ca wt/wt from CaCO_3_; *L*) and calcium-replete diet (0.55% Ca wt/wt from CaCO_3_; *S1*). Calcium-replete diet was prepared by adding CaCO3 into *L* to obtain a final of 0.55% w/w calcium (Supplemental Tables 1 and 2). Rats fed either *L* or *S1* exhibited an increase in bone growth as they got older from the age of 6 to 15 weeks. Comparing tibia and femur of the 15-week old to those of the 6-week old *S1* rats (Table 1), bone from the 15-week old rats showed increases in bone length, dry weight and ash weight, bone volume/tissue volume (BV/TV) and trabecular thickness (Tb.Th) as analyzed by bone histomorphometry, increase in mechanical property as analyzed by 3-point bending, and increases in cortical bone mineral density (BMD), cortical bone mineral content (BMC), cortical thickness (Ct.Th), cortical area (Ct.A) of metaphyseal and diaphyseal mid-shaft femur as analyzed by pQCT. However, trabecular BMD and BMC did not change with increase in age in *S1* reflecting the concurrent increase in the size of bone. On the other hand, comparing adolescent rats fed low calcium diet for 2 weeks (6 week old *L*) and young adult rats fed low calcium for 11 weeks (15 week old *L*), the 15-week old *L* showed normal bone length and increased in bone weight, trabecular BMC, cortical BMC, Ct.Th and Ct.A. Compared with corresponding *S1* control, bones of rats after 2 and 11 week calcium insufficiency exhibited drastic reductions in bone mechanical properties, cortical BMD, cortical BMC, Ct.Th, and Ct.A of metaphyseal and diaphyseal mid-shaft femur. Bone impairment found in rats fed low calcium for 11 weeks was less severe than those fed low calcium for 2 weeks. However, trabecular BMD was significantly decreased after 11 weeks but not after 2 weeks of low calcium diet. Magnitude of changes in bone microstructural parameters in *L* compared with corresponding *S1* after 11 weeks of low calcium diet were greater than those after 2 weeks of low calcium diet. Blood chemical analysis revealed that dietary calcium insufficiency for 2 and 11 weeks led to hypocalcemia (lower levels of total calcium and ionized calcium), lower level of 25(OH)D_3_, and higher levels of inorganic phosphate (only in 11 weeks) and hormone 1,25(OH)_2_D_3_ (Table 1).

**Table 1:** Tibial length, femur weight, 2D bone microstructure, bone mechanical properties, bone mineral density and 3D microstructure, and blood chemistry of 6-week and 15-weeks old rats fed low calcium diet for 2, and 11 weeks, respectively. *L*, low calcium diet; *S1*, calcium-replete diet with 25(OH)D_3_. *P < 0.05, **P < 0.01, ***P < 0.001 compared with corresponding *S1*, ^†††^*P* < 0.001 compared with 6-week-old with the same treatment.

	6-Week-old				15-Week-old			
	S1		L		S1		L	
	Mean ± SEM	*n*	Mean ± SEM	*n*	Mean ± SEM	*n*	Mean ± SEM	*n*
Bone length and weight								
Tibial length (cm)	3.139 ± 0.015	8	3.199 ± 0.042	8	3.780 ± 0.025^†††^	8	3.849 ± 0.028^†††^	8
Dry weight (g)	0.2889 ± 0.007	8	0.2150 ± 0.004***	8	0.6259 ± 0.019^†††^	8	0.4344 ± 0.009***^†††^	8
Ash weight (g)	0.1668 ± 0.004	8	0.0989 ± 0.001***	8	0.3865 ± 0.012^†††^	8	0.2116 ± 0.003***^†††^	8
2D bone microstructure								
BV/TV (%)	20.82 ± 0.803	8	10.61 ± 0.79***	6	31.98 ± 1.701^†††^	7	10.13 ± 1.359***	8
Tb.Th. (µm)	48.14 ± 1.055	8	30.10 ± 1.433***	6	66.83 ± 2.509^†††^	7	47.64 ± 2.988***^†††^	8
Tb.Sp. (µm)	185.1 ± 8.810	8	256.8 ± 10.88	6	144.0 ± 8.365	7	465.0 ± 52.63***^†††^	8
Tb.N. (mm^−1^)	4.329 ± 0.154	8	3.506 ± 0.121*	6	4.780 ± 0.167	7	2.101 ± 0.224***^†††^	8
Bone mechanical properties								
Maximal load (N)	60.44 ± 2.668	8	26.66 ± 1.963***	8	123.3 ± 10.23^†††^	8	67.33 ± 1.895***^†††^	8
Ultimate displacement (mm)	1.570 ± 0.082	8	2.147 ± 0.202**	8	0.774 ± 0.075^†††^	8	1.029 ± 0.058^†††^	8
Stiffness (N/mm)	124.9 ± 7.223	8	46.48 ± 4.353*	8	375.5 ± 38.13^†††^	8	178.2 ± 5.894***^†††^	8
Modulus (GPa)	5.354 ± 0.286	8	1.954 ± 0.142*	8	15.43 ± 1.540^†††^	8	7.539 ± 0.495***^†††^	8
Bone mineral density and content, and related parameters								
Metaphyseal distal femur								
Trabecular BMD (g/cm^2^)	0.3604 ± 0.007	8	0.3279 ± 0.048	8	0.3931 ± 0.007	8	0.1729 ± 0.018***^††^	7
Trabecular BMC (mg/mm)	4.131 ± 0.114	8	0.4775 ± 0.208***	8	3.471 ± 0.179	8	2.130 ± 0.181***^†††^	7
Cortical BMD (g/cm^2^)	0.8994 ± 0.005	8	0.7140 ± 0.031***	8	1.072 ± 0.009^†††^	8	0.954 ± 0.013***^†††^	7
Cortical BMC (mg/mm)	2.984 ± 0.130	8	0.098 ± 0.020***	8	9.145 ± 0.460^†††^	8	2.790 ± 0.156***^†††^	7
Cortical thickness (mm)	0.2384 ± 0.010	8	0.025 ± 0.003***	8	0.6053 ± 0.037^†††^	8	0.2099 ± 0.011***^†††^	7
Cortical area (cm^2^)	3.304 ± 0.135	8	0.1255 ± 0.026***	8	8.596 ± 0.475^†††^	8	2.908 ± 0.134***^†††^	7
Diaphyseal midshaft femur								
Cortical BMD (g/cm^2^)	1.172 ± 0.008	8	0.992 ± 0.007***	8	1.296 ± 0.004^†††^	8	1.145 ± 0.013***^†††^	7
Cortical BMC (mg/mm)	4.664 ± 0.093	8	2.043 ± 0.062***	8	7.746 ± 0.114^†††^	8	4.361 ± 0.068***^†††^	7
Cortical thickness (mm)	0.463 ± 0.008	8	0.231 ± 0.008***	8	0.554 ± 0.004^†††^	8	0.354 ± 0.007***^†††^	7
Cortical area (cm^2^)	3.981 ± 0.080	8	2.058 ± 0.056***	8	5.979 ± 0.088^†††^	8	3.813 ± 0.081***^†††^	7
Blood chemistry								
25(OH)D_3_ (ng/mL)	116.7 ± 3.321	6	44.79 ± 6.026***	6	81.43 ± 10.30	7	34.85 ± 3.572***	7
1,25(OH)_2_D_3_ (nM)	1.214 ± 0.105	7	1.712 ± 0.158*	7	1.396 ± 0.201	7	2.303 ± 0.086***	7
Total calcium (mM)	2.788 ± 0.070	8	2.459 ± 0.035***	8	2.724 ± 0.042	8	2.590 ± 0.033	8
Ionized calcium (mM)	1.298 ± 0.012	8	1.223 ± 0.011***	8	1.344 ± 0.006	8	1.221 ± 0.010***	8
Inorganic phosphate (mM)	3.823 ± 0.191	8	4.031 ± 0.064	8	2.823 ± 0.137	8	3.534 ± 0.094**	8

### Tuna bone calcium supplementation resulted in higher fractional calcium absorption and bone formation rate than calcium carbonate supplementation

**Figure 1:**
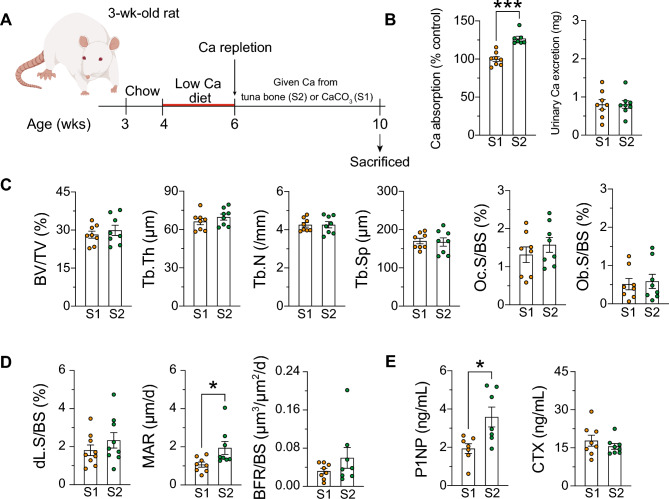
Tuna bone calcium supplementation resulted in higher fractional calcium absorption and bone formation rate than calcium carbonate supplementation. (**A**) Experimental design, all rats were challenged with low calcium diet (0.15% w/w) for 2 weeks, then diet was switch to calcium-replete diet (0.55% w/w) and fed for another 4 weeks. Extra calcium (0.4% w/w) came from tuna bone (*S2*) or CaCO_3_ (*S1*). (**B**) Intestinal fractional calcium absorption and 3-day urinary calcium excretion, (**C**) bone microstructure analyzed by bone histomorphometry (bone volume fraction, BV/TV, trabecular thickness, Tb.Th, trabecular separation, Tb.Sp, trabecular number, Tb.N, osteoclast surface normalized with bone surface, Oc.S/BS and osteoblast surface normalized with bone surface, Ob.S/BS), (**D**) dynamic parameters (doubled labeling surface normalized with bone surface, mineral apposition rate, MAR, bone formation rate normalized with bone surface, BFR/BS), and (**E**) bone turnover markers analyzed by commercial ELISA (bone formation marker, P1NP and bone resorption marker, CTX-1). *S1*, calcium supplementation in diet from CaCO_3_, *S2*, calcium supplementation in diet from tuna bone. Results are expressed as means ± SE. The different between two sets of data was determined by two-tailed unpaired Student’s *t* test. *P < 0.05, **P < 0.01, and ***P < 0.001 compared with CaCO_3_ (*S1*).

All rats were challenged with low calcium diet (*L*) for 2 weeks, thereafter, the effect of calcium supplementation from tuna bone (denoted as *S2*) was compared with calcium carbonate (denoted as *S1*) (Fig. 1A). Fractional calcium absorption in young rats fed extra calcium from tuna bone was significantly higher than that of rats fed CaCO_3_ (126.1 ± 3.15% in *S2* vs. 100.0 ± 3.15% in *S1*, *P* < 0.001, where mean of *S1* was normalized to 100%) and the extra absorbed calcium was not excreted into the urine (Fig. 1B). Rats fed tuna bone calcium and CaCO_3_ had similar body weight that increased with age from 6 to 10 weeks. Supplemental Table 4 showed no difference in tibial length or bone mechanical properties. pQCT analysis of the femoral metaphysis and mid-shaft diaphysis showed no difference in BMD or BMC of trabecular (Tb.BMD and Tb.BMC), cortical and sub-cortical region (Sub.Ct.BMD and Sub.Ct.BMC) or cortical compartment (Ct.BMD and Ct.BMC). Ct.Th, Ct.A, periosteal perimeter and endosteal perimeter (Ps.Pm and Es.Pm, respectively) were not different (Supplemental Table 4). Bone histomorphometry showed no difference in the microstructure, and osteoblast and osteoclast surface normalized with bone surface (Fig. 1C). However, rat fed calcium supplementation from tuna bone exhibited higher mineral apposition rate (2.331 ± 0.405 in *S2* and 1.806 ± 0.280 µm/day in *S1*, *P* < 0.05) and serum level of bone formation marker, procollagen type-1 N-terminal peptide (P1NP) (3.582 ± 0.522 in *S2* and 1.933 ± 0.256 ng/mL in *S1*, *P* < 0.05), without any change in serum level of bone resorption marker, C-terminal telopeptide of type 1 collagen (CTX) (15.54 ± 1.132 in *S2* and 17.81 ± 2.162 ng/mL in *S1*, *P* > 0.05) (Fig. 1D,E). Blood chemical analysis showed no difference in the levels of 25(OH)D_3_, 1,25(OH)_2_D_3_, and total calcium between these 2 treatments, whereas serum inorganic phosphate was lower in rats given tuna bone calcium supplementation (Supplemental Table 4).

### Calcium supplementation from tuna bone, tuna bone with tuna head oil added 25(OH)D_3_, and tuna bone with 25(OH)D_3_ enhanced fractional calcium absorption by upregulating serum 1,25(OH)_2_D_3_ and restoring serum calcium and inorganic phosphate to the levels of control

**Figure 2:**
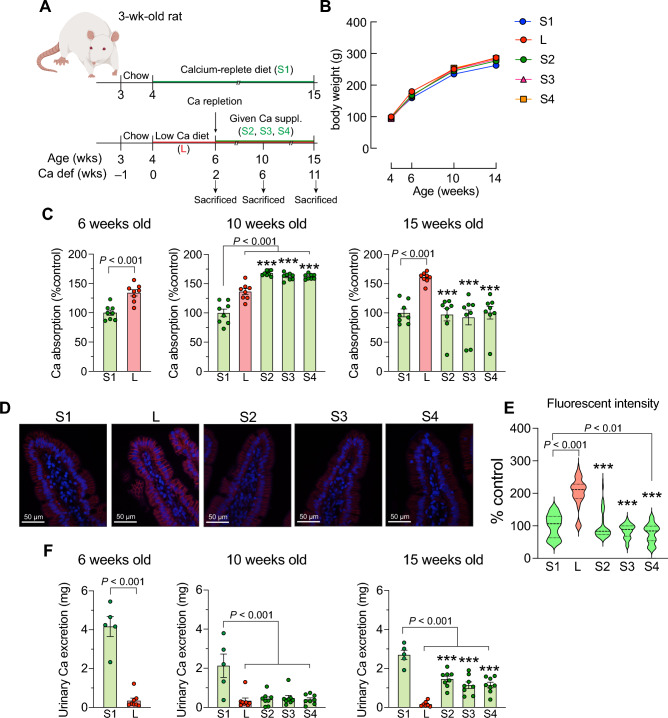
Calcium supplementation from tuna bone, tuna bone with tuna head oil and tuna bone with 25(OH)D_3_ enhanced fractional calcium absorption. (**A**) Experimental design, 4-week female rats were received calcium-replete diet (0.55% w/w as a CaCO_3_) and daily oral given 25(OH)D_3_ in the dose of 20 IU/kg until age of 15 weeks as normal baseline (denoted as *S1,* n = 8). Another set of 4-week female rats (n = 32), they were all challenged with low calcium diet (0.15% w/w) for 2 weeks, thereafter, rats were randomly divided into 4 groups, i.e., (i) stayed on low calcium diet (*L, n* = 8) or (ii) switched to calcium repletion diet (0.55% w/w) in which extra calcium was from tuna bone (*S2*, *n* = 8), (iii) tuna bone calcium repletion diet with tuna head oil added 25(OH)D_3_ (*S3*, *n* = 8) and (iv) tuna bone calcium repletion diet with commercial 25(OH)D_3_ (*S4, n* = 8). Thee-day calcium balance study was conducted in 3 time points, i.e., 2 weeks (baseline, before calcium supplement), 4 weeks and 11 weeks after calcium supplementation (animal were on age of 6, 10 and 15 weeks, respectively), (**B**) body weight, (**C**) relative fractional calcium absorption at rat’s age of 6, 10 and 15 weeks, (**D**) Representative images of immunostaining against PMCA protein, (**E**) quantitative data of fluorescent intensity of PMCA1 (relative expression to *S1*), and (**F**) 3-day urinary calcium excretion at rat’s age of 6, 10 and 15 weeks. Results are expressed as means ± SE. The different between two sets of data was determined by two-tailed unpaired Student’s *t* test. The differences between five experimental groups were determined by one-way ANOVA followed by Tukey post hoc test. ***P < 0.001 compared with *L*.

The timeline for age-matched rats fed with calcium-replete diet from CaCO_3_ (*S1*) is shown in Fig. 2A (upper panel) in which S1 rats received 0.55% w/w calcium throughout experimental period. Timeline for rats on low calcium diet (*L*) and rats receiving 3 formulae of tuna calcium supplements after 2 weeks of low dietary calcium diet is shown in Fig. 2A (lower panel). The diet ingredients were shown in Supplemental Tables 1 and 2. To find the optimal treatment duration, we performed a preliminary study in 4-week-old young growing female rats by investigating the alteration of femoral trabecular bone mineral density (BMD) by using an in vivo micro-computed tomography (Skyscan 1178). It showed that tuna bone calcium supplementation in 3 formulae for 4 and 9 weeks was able to completely restored bone loss to age-matched calcium-replete diet (*S1*). Therefore, we chose 9 weeks calcium supplementation for this study (Supplemental Fig. 2).

The three tuna bone calcium supplement formulae were tuna bone calcium (*S2*), tuna bone with tuna head oil added 25(OH)D_3_ (*S3*) and tuna bone with commercial 25(OH)D_3_ (*S4*). Tuna bone calcium supplement *S2* diet was prepared by mixing 0.4% w/w extra calcium from tuna bone powder into low calcium diet power (total calcium of 0.55% w/w). It showed that rats in groups *S2*, *S3* and *S4* had similar body weight to those of *L* and *S1* (Fig. 2B). The 6-week-old rats fed low calcium for 2 weeks during adolescence (4–6 week old) were found to upregulate the fractional calcium absorption that remained significantly higher than in the age-matched *S1* group when calcium insufficiency continued for another 4 or 9 weeks (133.9 ± 5.322% in 6-week *L*, 136.5 ± 4.952% in 10-week *L*, 160.5 ± 3.231% in 15-week *L*, where age-matched *S1* was normalized to 100%, the data was compared between blue bars and red bars in Fig. 2C). The 10-week-old rats that received 4 weeks of calcium supplementation of *S2*, *S3* and *S4* showed even higher fractional calcium absorption than those of *L* (Fig. 2C, middle panel). However, fractional calcium absorption returned to the basal level after 9 weeks of calcium supplementation, i.e., at 15 weeks of age (Fig. 2C, right panel). Immunostaining of PMCA1, a calcium transporter expressed at basolateral membrane of enterocytes, which is responsible for pumping calcium out of the cell, showed increase in the protein expression in *L* compared with *S1*, and the expression was compromised by calcium supplementation with or without 25(OH)D_3_ (Fig. 2D,E). As expected, urinary calcium excretion was significantly decreased in *L* (Fig. 2F). While 4 weeks of calcium supplementation did not change urinary calcium excretion in *L* (Fig. 2E, middle panel), 9-week calcium supplementation significantly increased the urinary calcium excretion in *L* by 10 folds (Fig. 2E, right panel).

Serum level of 25(OH)D_3_ was consistently lower in *L* compared with the age-matched *S1* (Table 2). Similarly, the 10-week-old *B* group show a significantly lower level of 25(OH)D_3_ when compared with *S1*. However, 25(OH)D_3_ level of the 15-week-old *S2* was not different from that of *S1*. Supplementation of tuna head oil added 25(OH)D_3_ in *S3* significantly increased the serum level of 25(OH)D_3_ in both 10- and 15-week old rats when compared with the corresponding *L* groups. Animals fed low calcium diet with or without calcium supplementation had higher serum level of 1,25(OH)_2_D_3_ at both 10 and 15 weeks of age compared with age-matched *S1*. Low calcium diet also resulted in hypocalcemia and hyperphosphatemia. Calcium supplementation from *S2*, *S3* and *S4* restored the level of ionized calcium and inorganic phosphate to the level seen in *S1*, but the total calcium levels of *S2* and *S4* were still lower than that of *S1* (Table 2).

**Table 2:** Femoral length and weight, serum level of 25(OH)D_3_, 1,25(OH)_2_D_3_, CTX, P1NP, total calcium, ionized calcium and inorganic phosphate of calcium-replete diet in which calcium came from CaCO_3_ (*S1*), rat fed with low calcium (*L*), rat received calcium supplementation from tuna bone (*S2*), tuna bone calcium supplement with tuna head oil added 25(OH)D_3_ (*S3*) or tuna bone calcium supplement with 25(OH)D_3_ (*S4*). *P < 0.05, **P < 0.01, ***P < 0.001 compared with *S1*, ^†^*P* < 0.05, ^††^ *P* < 0.01, ^†††^*P* < 0.001 compared with L.

	*n*	Mean ± SE				
		S1	L	S2	S3	S4
Femur						
Length (cm)	8	3.426 ± 0.032	3.518 ± 0.022	3.509 ± 0.035	3.494 ± 0.020	3.484 ± 0.013
Dry weight (g)	8	0.6259 ± 0.019	0.4344 ± 0.009***	0.6205 ± 0.020^†††^	0.6327 ± 0.016^†††^	0.6178 ± 0.020^†††^
Ash weight (g)	8	0.3865 ± 0.012	0.2116 ± 0.003***	0.3883 ± 0.011^†††^	0.3919 ± 0.011^†††^	0.3811 ± 0.013^†††^
25(OH)D_3_ (ng/mL)						
10 weeks	5–7	109.0 ± 7.688	40.92 ± 5.483***	61.36 ± 3.861**	99.09 ± 12.83^††^	N/A
15 weeks	6–7	81.43 ± 10.30	34.85 ± 3.572**	59.01 ± 10.69	91.98 ± 13.22^†††^	N/A
1,25(OH)_2_D_3_ (nM)						
10 weeks	7	0.950 ± 0.150	1.833 ± 0.100**	1.921 ± 0.129***	1.956 ± 0.138***	N/A
15 weeks	6–7	1.397 ± 0.201	2.303 ± 0.087**	2.086 ± 0.115*	2.343 ± 0.195***	N/A
CTX (ng/mL)						
10 weeks	7	14.94 ± 2.244	32.95 ± 2.210***	19.53 ± 2.718^†††^	18.83 ± 1.265^†††^	17.82 ± 1.925^†††^
15 weeks	6	11.24 ± 0.686	26.12 ± 3.049***	14.20 ± 2.751^†^	12.73 ± 1.572^††^	17.11 ± 2.061^†^
P1NP (ng/mL)						
10 weeks	7	2.720 ± 3.885	5.025 ± 0.562**	2.338 ± 0.353^††^	2.958 ± 0.228^†^	4.011 ± 0.499
15 weeks	7	1.177 ± 0.077	4.960 ± 0.863***	2.276 ± 0.353^††^	1.773 ± 0.256^†††^	1.631 ± 0.356^†††^
Total calcium (mM)	8	2.724 ± 0.043	2.590 ± 0.033	2.503 ± 0.059*	2.580 ± 0.045	2.513 ± 0.045*
Ionized calcium (mM)	8	1.344 ± 0.006	1.221 ± 0.009***	1.298 ± 0.017^†††^	1.286 ± 0.011^††^	1.281 ± 0.012^††^
Inorganic phosphate (mM)	8	2.823 ± 0.137	3.534 ± 0.094*	2.906 ± 0.273	2.916 ± 0.182	3.145 ± 0.100

Of note, bone weight, serum levels of 25(OH)D_3,_ 1,25(OH)_2_D_3,_ and blood chemistry (total calcium, ionized calcium and inorganic phosphate) in 15 weeks of *S1* and *L* groups were from the same data set shown in Table 1.

### Calcium supplementation from tuna bone, tuna bone with tuna head oil added 25(OH)D_3_ and tuna bone with 25(OH)D_3_ mitigated calcium insufficiency-induced osteoporosis

Femoral length of *L* group was not different from calcium-replete diet *S1*, suggesting that calcium insufficiency in this study did not impair bone linear growth. Rats fed tuna bone calcium supplement from 3 formulae also had similar bone length to *L* and *S1* (Table 2). Bones from *L* had lower dry and ash weight indicating lower mineral content. Bone weight after calcium supplementation was fully restored to that of *S1* (Table 2). Representative coronal images of the micro-computed tomography scanning of femur at the age of 10 weeks and 15 weeks, i.e., after 4 weeks (upper panel) and 9 weeks of calcium supplementation (lower panel) are shown in Supp Fig. 1. At the age of 10 weeks, *L* exhibited a drastically reduced metaphyseal trabecular bone area and mid-shaft cortical shell, whereas calcium supplementation in *S2*, *S3* and *S4* groups resulted in greater trabecular bone area and thickening of the mid-shaft cortical shell. At the age of 15 weeks, bones of every group were longer than those of the 10-week-old animals and the effects of calcium supplementation were similar to those seen in the 10 week old animals (Supp Fig. 1).

**Figure 3:**
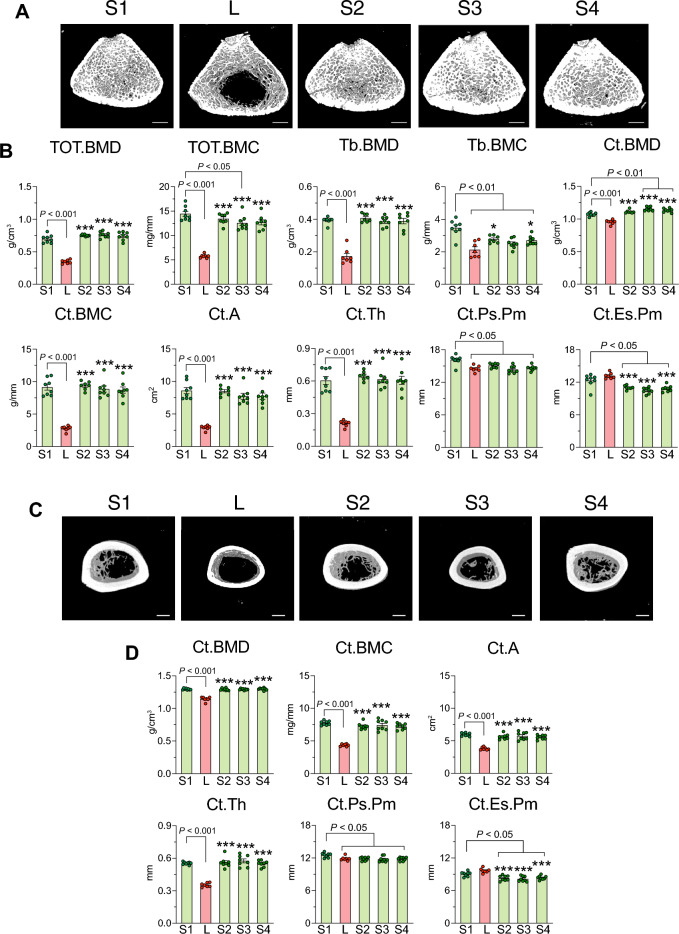
Calcium supplementation from tuna bone, tuna bone with tuna head oil added 25(OH)D_3_ and tuna bone with 25(OH)D_3_ mitigated calcium insufficiency-induced osteoporosis. Four-week female rats were received calcium-replete diet (0.55% w/w as a CaCO_3_) and daily oral given 25(OH)D_3_ in the dose of 20 IU/kg until age of 15 weeks as normal baseline control (denoted as *S1*, n = 8). Another set of 4-week female rats (n = 32), they were all challenged with low calcium diet (0.15% w/w) for 2 weeks, thereafter, rats were randomly divided into 4 groups, i.e., (i) stayed on low calcium diet (*L, n* = 8) or (ii) switched to calcium repletion diet (0.55% w/w) in which extra calcium was from tuna bone (*S2, n* = 8), (iii) tuna bone calcium repletion diet with tuna head oil added 25(OH)D_3_ (*S3, n* = 8) and (iv) tuna bone calcium repletion diet with commercial 25(OH)D_3_ (*S4, n* = 8). (**A**) Representative images of three-dimensional reconstruction of metaphyseal distal femur (cross-sectional view), Scale bars, 1 mm, (**B**) bone mineral density and content (BMD and BMC, respectively) of total bone (TOT), trabecular (Tb) and cortical compartment (Ct). Cortical area (Ct.A) thickness (Ct.Th), periosteal perimeter (Ct.Ps.Pm) and endosteal perimeter (Ct.Es.Pm), all parameters were analyzed by pQCT at distal metaphyseal femur, (**C**) representative images of three-dimensional reconstruction of mid-shaft diaphyseal femur (cross-sectional view), Scale bars, 1 mm, and (**D**) cortical bone parameters analyzed by pQCT at mid-shaft femur. Results are expressed as means ± SE. The differences between five experimental groups were determined by one-way ANOVA followed by Tukey post hoc test. *P < 0.05, ***P < 0.001 compared with *L*.

**Figure 4:**
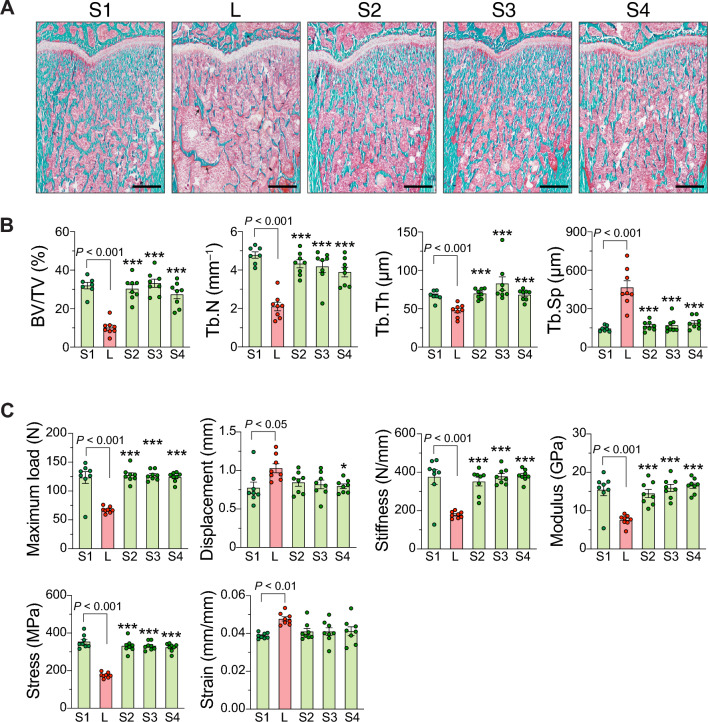
Calcium supplementation from tuna bone, tuna bone with tuna head oil added 25(OH)D_3_ and tuna bone with 25(OH)D_3_ improved bone microstructure impairments and mechanical properties. Experimental design was similar to Fig. 3. (**A**) Representative images of bone section stained with Goldner’s trichrome, Scale bars, 1 mm, (**B**) static bone parameters at proximal metaphyseal tibia analyzed by bone histomorphometry, and (**C**) mechanical properties analyzed by 3-point bending apparatus. Results are expressed as means ± SE. The differences between five experimental groups were determined by one-way ANOVA followed by Tukey post hoc test. *P < 0.05, ***P < 0.001 compared with *L*.

Representative scanning images of the cross-sectional section of metaphyseal distal femur were shown in Fig. 3A. BMD and BMC of total tissue (TOT; including trabecular and cortical compartments), trabecular and cortical compartments in *L* were drastically reduced when compared with *S1*. As seen in Fig. 3B, Ct.A and Ct.Th were also lower than those of *S1*. The thinning of Ct.Th and Ct.A in *L* apparently resulted from reduction in Ct.Ps.Pm without any change in Ct.Es.Pm. All 3 tuna bone calcium supplement formulae (i.e., *S2*, *S3* and *S4*) appeared to restore bone microstructural parameters toward those of *S1*, i.e., TOT.BMD, TOT.BMC, Tb.BMD, Tb.BMC, Ct.BMD. Ct.BMC, Ct.A, and Ct.Th with an exception of Ct.Ps.Pm and Ct.Es.Pm. However, Tb.BMC of *S2*, *S3* and *S4* were still significantly lower than *S1*, while Ct.BMD of *S3* and *S4* was significantly higher than that of *S1* (Fig. 3B). Figure 3C depicted scanning images of the cross-sectional sections of femoral midshaft diaphysis. As expected, *L* had lower Ct.BMD, Ct.BMC, Ct.A, Ct.Th and Ct.Ps.Pm, and calcium supplementation from *S2*, *S3* and *S4* restored these parameters to those of *S1* (Fig. 3D). Even though the cortical shell of both femoral metaphysis and diaphysis was thicker after calcium supplementation, bone growth in width was compromised as Ct.Ps.Pm and Ct.Es.Pm were significantly lower than those of *S1* (Fig. 3B,D). Representative bone sections stained with Goldner’s trichrome were depicted in Fig. 4A. Bone microstructure analysis by bone histomorphometry showed *L* having a 68% reduction in bone volume fraction (BV/TV), reduction in Tb.N and Tb.Th, and increase in Tb.Sp. Tuna bone calcium supplementation from *S2*, *S3* and *S4* completely restored all values to the levels of *S1* (Fig. 4B). *L* exhibited a drastic impairment in mechanical property as shown in the reductions in maximum load, stiffness, Young’s modulus, and stress at maximum load, while an increase in maximum displacement and strain at maximum load. Calcium supplementation from *S2*, *S3* and *S4* returned the maximum load, stiffness, Young’s modulus and stress to the *S1* levels. All three formulae of calcium supplementation were able to lower the maximum displacement from the level seen in *L*, but only the reduction in *S4* was significantly different from that of *L*. Changes in strain showed a similar trend to that of maximum displacement (Fig. 4C).

### Voluntary running exercise improved bone mineral density and mechanical properties in calcium insufficiency-induced osteoporosis

**Figure 5:**
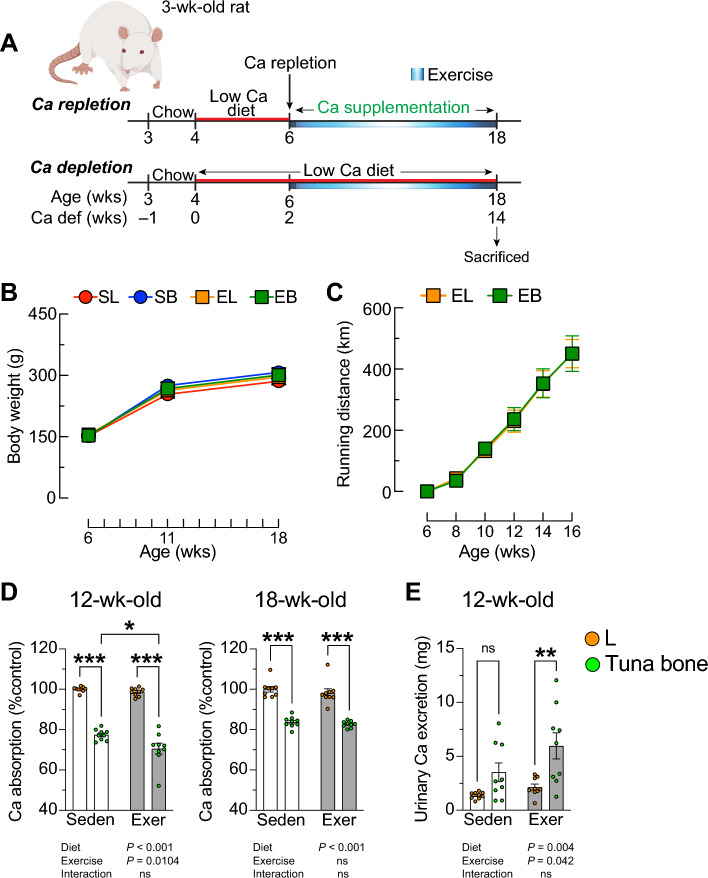
Calcium supplementation suppressed fractional calcium absorption and increased urinary calcium excretion in both sedentary and exercise groups. (**A**) Experimental design, 4-week female rats were challenged with low calcium diet for 2 weeks, thereafter, rats were randomly divided into 2 sets, calcium repletion diet (0.55% w/w) in which extra calcium was from tuna bone or stayed on low calcium diet (0.15% w/w; *L*) until being sacrificed in the age of 18 weeks. Each set, rats were randomly sub-divided into 2 groups, voluntary running exercise (rats were housed in cage-equipped with running wheel for 12 weeks) or sedentary (rats were housed in cage-equipped with running wheel but wheel was locked for 12 weeks), (**B**) body weight, (**C**) cumulative running distance, (**D**) relative fractional calcium absorption at rats on age of 12 weeks and 18 weeks, and (**E**) 3-day urinary calcium excretion. *SL*, sedentary with low calcium diet, *SB*, sedentary with calcium supplementation, *EL*, exercise with low calcium diet, *EB*, exercise with calcium supplementation. Results are expressed as means ± SE. Statistical analysis was conducted using two-way ANOVA with diet (*L* or *tuna* *bone* *calcium* *supplement* *diet*) and exercise (sedentary or exercise) as between-subject factors, followed by Tukey’s multiple comparisons posthoc test. *P < 0.05, **P < 0.01, and ***P < 0.001.

The experimental design to evaluate the effect of impact exercise and calcium supplementation on calcium and bone metabolism was shown in Fig. 5A. Animals that performed voluntary running exercise with calcium supplementation from tuna bone (*EB*) showed the same body weight gain as those of the exercise without supplementation group (*EL*), and the sedentary with and without calcium supplementation groups (*SB* and *SL*, respectively) (Fig. 5B). *EL* and *EB* also demonstrated similar exercise performance as shown by cumulative running distance (Fig. 5C). Similar to the experimental design in the first part, in this experiment, 12-week and 18-week old groups received calcium supplementation for 6 weeks and 12 weeks, respectively (Fig. 5D, left and right panels). Tuna bone supplemented *B* groups exhibited lower fractional calcium absorption compared to age-matched control groups whether they were sedentary or voluntary exercise group (70.598 ± 2.725% in *EB*, 98.670 ± 0.683% in *EL*, 77.409 ± 0.913% in *SB*, *P* < 0.001, % fractional calcium absorption showed as a relative change to mean value of *SL*, where mean value of *SL* was normalized to 100%). In the 12 week old group, exercise had no effect on fractional calcium absorption in calcium insufficient *L* group, whereas in *B* group that were fed adequate calcium diet, exercise significantly decreased calcium absorption (70.598 ± 2.725 in *EB* and 77.409 ± 0.913 in *SB*, *P* < 0.05, Fig. 5D, left). As for the 18 week old groups, exercise had no effect on fractional calcium absorption whether with or without calcium supplementation (Fig. 5D, right). Calcium supplementation had a tendency to increase urinary calcium excretion in sedentary group, but the increase reached a statistical significance only in the exercise group (5.973 ± 1.213 in *EB* and 2.148 ± 0.283 in *EL* (unit in mg over 3-day balance study, Fig. 5E).

**Figure 6:**
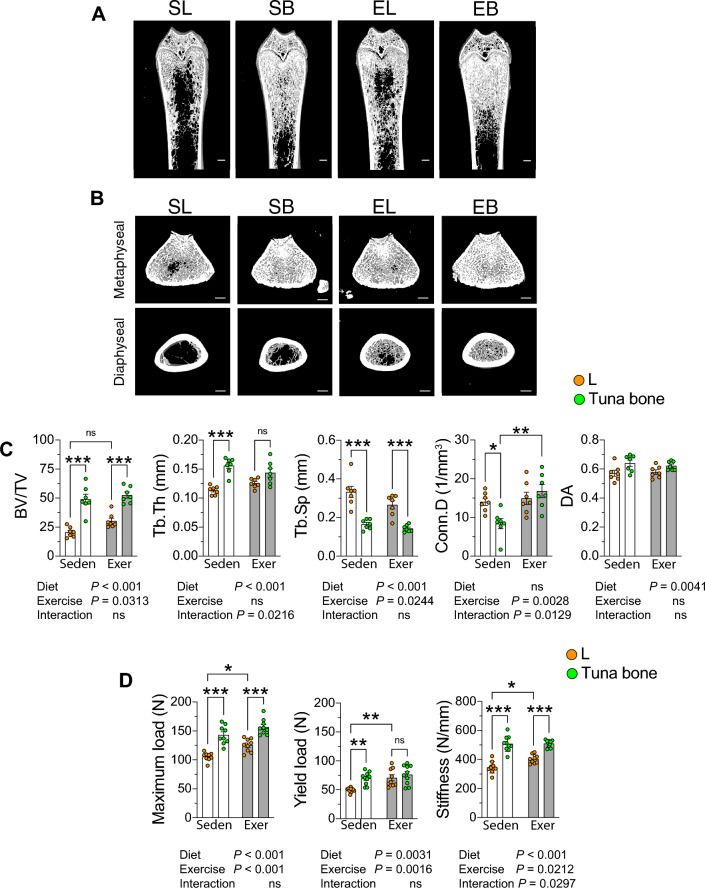
Voluntary running exercise improved bone microstructure and mechanical property in calcium insufficiency-induced osteoporosis. Experimental design was similar to Fig. 5. (**A**) Representative images of three-dimensional reconstruction of metaphyseal distal femur (longitudinal view), (**B**) representative images of three-dimensional reconstruction of metaphyseal distal femur (upper panels) and diaphyseal mid-shaft femur (lower panels), Scale bars, 1 mm, (**C**) bone microstructure analyzed by ultra-high resolution micro-computed tomography at distal femur, (**D**) mechanical properties analyzed by 3-point bending apparatus at femoral mid-shaft. Results are expressed as means ± SE. Statistical analysis was conducted using two-way ANOVA with diet (*L* or *tuna bone calcium supplement* diet) and exercise (sedentary or exercise) as between-subject factors, followed by Tukey’s multiple comparisons post-hoc test. *P < 0.05, **P < 0.01, and ***P < 0.001.

Representative 3D images of CT scanning of distal femur (longitudinal section) was shown in Fig. 6A, and cross-sectional section images of distal metaphyseal- and mid-shaft diaphyseal femur of *SL*, *SB*, *EL* and *EB* were shown in Fig. 6B. Neither calcium supplementation from tuna bone nor running exercise had effect on bone length (Table 3). On the other hand, it was clearly shown that calcium supplementation with or without exercise increased bone weight and improved bone microstructure by alleviating all calcium insufficiency-associated osteoporotic features. Interestingly, running exercise without calcium supplementation (*L*) significantly increased Tb.BMD, Ct.BMD and Ct.Th of the diaphyseal mid-shaft femur. Neither calcium supplement nor exercise had effect on Ct.BMD of metaphyseal region. The overall data from pQCT strongly suggested that calcium supplementation had greater benefits to bone than exercise. Result from two-way ANOVA indicates that calcium supplement had effect on both metaphyseal and diaphyseal midshaft, while exercise had effect on bone predominately at the diaphyseal midshaft. Moreover, the effect of calcium supplementation and exercise had interaction on Ct.BMD of diaphyseal midshaft. Data on blood chemical analysis showed that calcium supplementation in sedentary group, but not in the exercise group, significantly increased serum levels of total calcium and inorganic phosphate (Table 3). From the ultra-high resolution µCT analysis (Fig. 6C), it was found that calcium supplementation increased BV/TV, while decreasing Tb.Sp in both sedentary and exercise groups. *SB* exhibited a higher Tb.Th than *SL*. Calcium supplementation resulted in a lower trabecular connectivity density (Conn.D) in sedentary (8.390 ± 1.305 in *SB* vs. 14.046 ± 0.928 in *SL*, *P* < 0.05), but not in exercise groups (16.804 ± 1.682 in *EB* vs. 14.939 ± 1.566 in *EL*, *P* > 0.05). Calcium supplementation with exercise increased Conn.D more than calcium supplement without exercise (16.804 ± 1.682 in *EB* vs. 8.390 ± 1.305 in *SB*, *P* < 0.01). Neither calcium supplementation nor exercise had effect on the degree of anisotropy. From the mechanical property analysis, exercise was found to significantly increase these mechanical parameters only in calcium deficient *L* groups, but not in calcium supplemented B groups. In contrast, tuna bone calcium supplementation, whether with or without exercise, significantly increased the maximum load and stiffness, and increased yield load in the sedentary group (Fig. 6D).

**Table 3:** Bone length, bone weight, bone mineral density and content, and 3-dimensional microstructure of femur and blood chemistry of sedentary and voluntary running exercise fed low calcium diet or calcium supplementation from tuna bone. *P < 0.05, **P < 0.01, ***P < 0.001 compared with corresponding *L*, ^†^*P* < 0.05 compared with sedentary *L*. Significant values are in bold.

	*n*	Sedentary		Exercise		P value		
		SL	SB	EL	EB	Supplement	Exercise	Interaction
Bone length								
Femur (cm)	10	3.477 ± 0.031	3.475 ± 0.020	3.479 ± 0.013	3.525 ± 0.035	0.4092	0.3303	0.3683
Tibia (cm)	10	3.897 ± 0.018	3.873 ± 0.030	3.890 ± 0.017	3.921 ± 0.013	0.8684	0.3348	0.1981
Bone weight								
Dry (g)	10	0.583 ± 0.013	0.692 ± 0.019***	0.603 ± 0.012	0.710 ± 0.016***	**< 0.0001**	0.2330	0.9702
Ash (g)	10	0.334 ± 0.011	0.446 ± 0.015***	0.363 ± 0.010	0.455 ± 0.011***	**< 0.0001**	0.1117	0.4011
Metaphyseal distal femur								
Subcortical BMD (g/cm^3^)	10	0.963 ± 0.024	0.964 ± 0.032	0.977 ± 0.047	0.962 ± 0.038	0.5449	0.6036	0.4893
Subcortical BMC (mg/mm)	10	7.138 ± 0.767	15.78 ± 3.134c	7.967 ± 1.140	17.416 ± 2.720***	**< 0.0001**	0.0828	0.5621
Trabecular BMD (g/cm^3^)	10	0.266 ± 0.045	0.448 ± 0.037***	0.317 ± 0.048^**†**^	0.476 ± 0.015***	**< 0.0001**	**0.0028**	0.3438
Trabecular BMC (mg/mm)	10	3.042 ± 0.562	1.668 ± 0.653***	3.403 ± 0.757	1.353 ± 0.465***	**< 0.0001**	0.9071	0.0928
Trabecular area (cm^2^)	10	11.27 ± 0.984	3.848 ± 1.729***	10.63 ± 1.738	2.88 ± 1.049***	**< 0.0001**	0.0828	0.7140
Cortical BMD (g/cm^3^)	10	1.102 ± 0.018	1.100 ± 0.040	1.073 ± 0.032	1.064 ± 0.036	0.5834	**0.0035**	0.7274
Cortical BMC (mg/mm)	10	6.118 ± 0.581	13.79 ± 3.142***	6.938 ± 0.963	15.28 ± 2.602***	**< 0.0001**	0.0932	0.6207
Cortical area (cm^2^)	10	5.561 ± 0.536	12.96 ± 3.076***	6.182 ± 0.855	14.46 ± 2.535***	**< 0.0001**	0.1121	0.5048
Cortical thickness (mm)	10	0.396 ± 0.032	1.022 ± 0.267***	0.451 ± 0.060	1.141 ± 0.195***	**< 0.0001**	0.1118	0.5514
Cortical Ps.Pm (mm)	10	15.30 ± 0.560	15.92 ± 0.820	15.48 ± 1.097	16.21 ± 1.014	**0.0233**	0.4097	0.8454
Cortical Es.Pm (mm)	10	12.81 ± 0.520	9.495 ± 1.300***	12.65 ± 1.019	9.040 ± 0.997***	**< 0.0001**	0.3335	0.6478
Diaphyseal midshaft femur								
Cortical BMD (g/cm^3^)	10	1.234 ± 0.017	1.305 ± 0.012***	1.258 ± 0.022^**†**^	1.305 ± 0.011***	**< 0.0001**	**0.0230**	**0.0251**
Cortical BMC (mg/mm)	10	6.434 ± 0.461	7.967 ± 0.919***	7.168 ± 0.512	8.082 ± 0510*	**< 0.0001**	**0.0396**	0.1282
Cortical area (cm^2^)	10	5.219 ± 0.396	6.107 ± 0.718**	5.701 ± 0.465	6.194 ± 0.404	**< 0.0001**	**0.0390**	0.1202
Cortical thickness (mm)	10	0.495 ± 0.027	0.569 ± 0.055**	0.551 ± 0.044^**†**^	0.588 ± 0.034	**< 0.0001**	**0.0067**	0.1597
Cortical Ps.Pm (mm)	10	12.10 ± 0.465	12.50 ± 0.458	12.07 ± 0.456	12.38 ± 0.341	**0.0145**	0.6114	0.7075
Cortical Es.Pm (mm)	10	8.987 ± 0.456	8.923 ± 0.286	8.613 ± 0.502	8.682 ± 0.343	0.9883	**0.0196**	0.6016
Blood chemistry								
Total calcium (mM)	10	2.549 ± 0.031	2.686 ± 0.032*	2.476 ± 0.041	2.527 ± 0.031	**0.0088**	**0.0016**	0.2131
Ionized calcium (mM)	10	1.343 ± 0.016	1.324 ± 0.007	1.306 ± 0.007	1.304 ± 0.014	0.3805	**0.0212**	0.4769
Inorganic phosphate (mM)	10	2.540 ± 0.092	2.906 ± 0.057*	2.559 ± 0.080	2.849 ± 0.086	**0.0002**	0.8134	0.6374

## Discussion

### Adequate calcium and 25(OH)D_3_ intake during childhood and adolescent determine peak bone mass

Low dietary calcium intakes and poor 25(OH)D_3_ status are common findings in children living in developing countries. Low dietary calcium intakes are typically observed as a consequence of a diet deficient in dairy products and high in phytates and oxalates which reduce calcium bioavailability. Childhood and adolescence are the critical period of bone development and mineralization, and are the period for maximizing genetically predetermined peak bone mass. Even though genetic predisposition determines up to 80% of peak bone mass^23^, environmental factors are considered to be important modulators of an individual's genetic potential, such as nutrition and physical exercise especially in late childhood and early puberty^23–28^. Peak bone mass and subsequent bone losses are important determinants of osteoporosis later in life. Thus, maximizing peak bone mass in early life, a period of relatively high plasticity of the skeleton in response to physical forces, is advocated as a way to protect against osteoporotic fractures later in life. It was shown that daily calcium intake was associated with mineral acquisition in adolescents in a dose response manner^29^. Thus, inadequate calcium intake during this critical period could reduce bone accrual and impede bone growth, which, in turn, reduced bone size. This study aimed to investigate whether calcium supplementation as well as impact exercise was able to ameliorate bone defects caused by inadequate calcium intake in young growing rats. According to the previous literatures, 4–6 weeks old in rats is considered young adult, which is a proxy of adolescence in human^30,31^. Creedon and Cashman^32^ showed in young growing female rats fed low calcium diet between aged of 5–8 weeks that 5-week-old rats fed 0.2% w/w calcium for 3 weeks resulted in a decreased femoral calcium content and increased urinary pyridinoline and deoxypyridinoline, both of which are bone resorption markers. Therefore, 4-week old young growing female rats was used in this study and was challenged with 0.15% w/w calcium diet to induce negative calcium balance.

### Inadequate calcium intake during adolescence and young adulthood results in a lower bone mass and compromises bone mechanical properties

Herein, we demonstrated that the body could undergo significant adaptation to low calcium intake by reducing renal calcium excretion and increasing fractional intestinal calcium absorption through enhanced 1,25(OH)_2_D_3_ production. Generally, bone mass and strength increased with age^33^. As expected, the 15-week-old rats in the present study had higher bone mass at both cortical and trabecular regions compared to the 6-week old rats (Table 1). At the site of trabecular metaphysis, higher bone mass was detected by bone histomorphometry but not pQCT technique, which was probably due to the lower sensitivity of the pQCT technique. Two weeks of dietary calcium insufficiency led to a drastic reduction in trabecular BMC, cortical BMD and cortical BMC in both adolescent and young adult rats. The reduction in trabecular BMC with no change in trabecular BMD in the adolescent 6-week-old rats was probably due to a parallel decrease in bone size and trabecular bone as shown by lower dry weight and ash weight (Table 1). The 15-week old young adult rats that remained on low calcium diet for another 11 weeks showed clear impairment of bone growth as seen in all bone-related parameters (except for bone length). Considering the impact of 2 and 11 weeks of low calcium diet on 6- and 15-week old rats, respectively, on trabecular bone, reduction in trabecular bone volume in 6-week old low calcium rats was due mostly to decrease in trabecular thickness. On the other hand, the drastic reduction in bone volume in 15-week old low calcium rats was due to a decrease in trabecular thickness as well as a huge reduction in trabecular number. This study confirmed previous reports that inadequate calcium intake did not impair bone growth in length in young growing female rodents^34^, young adult male rodents^35^, and prepuberty girls^36^. However, it did impair the microscopic structure of bone that clearly compromise the mechanical properties and function.

### Calcium supplements derived from tuna bone exhibits a higher calcium bioavailability than CaCO_3_

Calcium supplements derived from natural sources are generally considered more beneficial than purified calcium carbonate from inorganic sources^37–40^. There are two important advantages for tuna bone as a calcium source. First, calcium in tuna bone is a form of hydroxyapatite (providing both calcium and phosphorus in an appropriate or optimal ratio), the nanocrystal of which is naturally formed in skeletal tissues. Second, certain nutrients in natural products present in bone have been shown to improve the calcium absorption. For example, collagen in bone can enhance calcium absorption as shown in the in vivo^37^ and in vitro experiments^38^. Calcium in the form of protein complex is also easily dissolved and released by protein digestion in the stomach rendering it ready for absorption in the small intestine. In agreement with our previous study, fish bone calcium supplement exhibited greater bioavailability than calcium carbonate^16^. Unlike with CaCO_3_ supplement, the higher fractional calcium absorption induced by tuna bone supplements did not lead to the extra absorbed calcium being excreted in the urine, but was retained to form new bone as shown by higher mineral apposition rate and bone formation marker (Fig. 1). However, we failed to observe any difference in bone static microstructure parameters after 4-week supplementation, only dynamic change, i.e., higher mineral apposition rate, was observed. Longer supplementation could have more pronounced effects on bone structure.

### Calcium supplementation from tuna bone with and without 25(OH)D_3_ mitigates negative calcium balance and restores bone defects caused by inadequate calcium intake

Herein, we showed that calcium supplementation from tuna bone did not suppress 1,25(OH)_2_D_3_ production, nor did it compromise intestinal calcium absorption. It further enhanced the fractional calcium absorption and mitigated negative body calcium balance as shown by the restoration of serum ionized calcium and almost all bone-related parameters to the control levels (Figs. 2, 3, 4 and Table 2). We found a modest additive effect of calcium supplementation together with 25(OH)D_3_ on cortical BMD of the distal femur as compared to the effect of calcium supplementation alone (Fig. 3A), but failed to observe any additive effect on intestinal calcium absorption or other bone parameters (Figs. 2, 3, 4). Although, 25(OH)D_3_ supplementation with tuna head oil increased the serum level of 25(OH)D_3_, but the level of hormone 1,25(OH)_2_D_3_ did not change (Table 2). This was possibly due to fact that calcium supplementation alone was able to effectively correct hypocalcemia so that there was no stimulator to enhance conversion of 25(OH)D_3_ to 1,25(OH)_2_D_3_. Therefore, we did not detect any change in serum 1,25(OH)_2_D_3_ level in the group receiving calcium supplement with 25(OH)D_3_ in S3 group.

Interestingly, calcium supplementation with or without 25(OH)D_3_ restored trabecular and cortical BMD, cortical thickness and cortical area to the normal values, but did not restore trabecular BMC, or cortical periosteal and endosteal perimeters of distal femur and midshaft femur (Fig. 3). The mechanical properties were also restored to normal level through compensatory increases in cortical thickness and area (Figs. 3 and 4). These findings indicate that transient inadequate calcium intake during childhood potently compromised bone accrual and bone growth in width, and these bone defects were long-lasting and could not be restored even with calcium supplementation during the period of adolescence to young adulthood. Our results were in partial agreement with the previous study in rats which showed that low calcium intake through adolescence had a nonreversible, deleterious effect on peak bone mass, i.e., rats fed inadequate calcium diet during childhood and adolescence (i.e., 4–12 weeks of age in rats) had lower peak bone mass even with the diet being switched to one with adequate calcium throughout adulthood till the age of peak bone mass (age of 37 weeks in rats)^41^. As mention earlier, dietary calcium intake was not directly associated with bone length, but rather controlled by a complex interaction of systemic and local factors, i.e., growth hormone, IGF-1, thyroid hormone, glucocorticoids and sex hormones^42–45^. An impairment of bone growth in width was highly significant because for bones of the same length, if one was twice as wide as the other, that bone would be eight times stronger^43^. Although calcium supplementation and the resulted increase in cortical thickness could restore bone strength to a normal level, its smaller diameter may make it more susceptible to fracture if exposed to risks brought on by inadequate calcium intake or menopause later in life.

It is well accepted that impact exercise or mechanical loading promoted osteogenesis, bone angiogenesis and increase in bone strength in both rodents and human^46–50^. Physical activity during early childhood and adolescence appears to be an important predictor of peak bone mass which accounts for up to 17% of the variance in BMD between individuals in their late 20s^27^. Moreover, aerobic exercise was reported to upregulate serum 1,25(OH)_2_D_3_ and intestinal calcium absorption in young human and rodents^51,52^ but was found to have no effect on calcium absorption in another human study^53^. In the present study, we found that 6 week running exercise in 6 week old rats led to a slight, but significant reduction in fractional calcium absorption when rats got to 12 weeks of age if they had received tuna bone calcium supplement, but not if they received low calcium diet. However, this suppressive effect was not observed if running protocol continued for another 6 weeks (Fig. 5). Thus, the effect of aerobic exercise on intestinal calcium absorption varied with body calcium status and exercise duration.

We further demonstrated that voluntary running exercise increased both trabecular and cortical BMD, cortical thickness, and improved mechanical properties in low calcium intake condition, but not in adequate calcium condition (Table 3 and Fig. 6). The benefit of exercise is usually region specific, i.e., at loaded site, which results from a local response to stress induced by loading. In contrast, the effect of calcium supplementation is more generalized and not restricted to a particular region^36^. Herein, we used femur and tibia to evaluate the possibility of combined benefits of exercise and calcium supplementation (Fig. 6, Table 3). We found that the effect of running exercise was predominately seen on cortical-related parameters, which may be explained by the fact that exercise increased periosteal bone formation with no effect on endosteal bone formation^54^. Calcium supplement on the other hand, benefited both trabecular metaphysis and diaphysis. We failed to see additive effect of exercise and calcium supplementation on bone-related parameters. It appeared that, tuna bone calcium supplementation alone was enough to alleviate the negative calcium balance in low calcium-fed group and maximized bone accrual in young adults. Bass and co-workers in a randomized controlled trial in 7–11-year-old boys, found some additive effects of exercise and calcium supplement on BMC of femur, whereby calcium supplement alone or exercise alone showed only a tendency to increase femoral BMC. Furthermore, this additive effect was region specific, i.e., increased femoral BMC but not tibia-fibula BMC even though both are loaded bone^55^. Similar finding was also report in randomized controlled trial study in girls^36^.

In partial agreement with the previous study in children^56^ which reported an interaction between calcium supplementation and physical activity in cortical bone (i.e., cortical area and thickness), this study found such interaction in cortical BMD at both metaphyseal and diaphyseal regions (Table 3). It was unclear whether the exercise-induced bone benefits during adulthood were retained and could reduce fracture risk in old age. Studies in human found that soccer players had higher BMD that was maintained several years after retirement from the sport. However, when they aged over 60 years, the fracture incidence in elderly former soccer players was not different from that of control^57^. Similar result was also found in young rat study^58^. On the contrary, other studies reported that exercise in youth was associated with a greater quality and strength in bones at older age^59,60^. Thus, long-term benefits of exercise in youth on the prevention of osteoporotic later in life is still debatable.

In conclusions, inadequate calcium intake during childhood and adolescence caused impairments of bone calcium accrual and appositional bone growth (bone growth in diameter), but did not impair linear bone growth, and this impairment persisted into young adulthood in rats. Calcium supplementation from a natural source such as tuna bone had greater bioavailability than that of calcium carbonate. Tuna bone calcium supplement with tuna head oil containing 25(OH)D_3_ and Omega-3 did not show any additive effect on fractional calcium absorption nor bone-related parameters compared with calcium supplement alone. In addition, both calcium supplementation from tuna bone and impact exercise (i.e., running) alleviated bone defects caused by low dietary calcium intake. Although, the present study provides supportive evidence for future use of calcium supplements from natural sources and exercise as therapeutic approaches or intervention for ameliorating bone impairment caused by low calcium intake, the data were mainly from animal studies. Therefore, these approaches need to be confirmed in young adolescent volunteers who consume relatively low calcium intake before being translated for clinical uses. Moreover, whether the beneficial outcomes of calcium supplementation together with exercise last until late adulthood or disappear after cessation of such intervention requires future investigations in both animals and human subjects.

## Materials and methods

### Animals

Three-week old Sprague–Dawley female rats were purchased from Nomura Siam International Co. Ltd, Bangkok, Thailand. They were housed in a controlled environment of 25 ± 2 °C, on a 12:12-h light–dark cycle with an average illumination of 200 lx for 7 days before experiment. Animals were fed with low calcium diet (0.15% w/w) containing 0.9% phosphorus, and 800 IU/kg 25(OH)D_3_ or calcium-replete diet (0.55% w/w) containing 0.9% phosphorus, and 800 IU/kg 25(OH)D_3_. Low calcium diet (CE-2; calcium presented as a CaCO_3_) was purchased from CLEA, Japan. Calcium-replete diet (or calcium supplement) was made by adding an extra 0.4% w/w calcium in low calcium diet powder (total calcium of 0.55% w/w). The extra calcium came from either calcium carbonate (*S1*) or from tuna bone powder (*S2*). As for low calcium diet (*L*) used in this experiment, the kibbles were produced without adding any extra calcium and served as the control group. Food ingredients of *L*, *S1* and *S2* were analyzed based on AOAC (2012) method (Supplemental Tables 1 and 2). As for another series of exercise experiment, 4-week female rats were individually housed in a cage-equipped with running wheel for 12 weeks (model 80859, Lafayette Instrument Company, Lafayette, IN, USA) in a controlled environment of 25 ± 2 °C, on a reversed 12:12-h light–dark cycle. Animal were fed ad libitum with free access to reverse osmosis (RO) water. The detail of experimental designs and animal groups were described in following sections. At the end of the study, animals were euthanized with intraperitoneal injection of 15 mg/kg xylazine (Thai Meiji Pharmaceutical Co., Ltd., Bangkok, Thailand) and 120 mg/kg zoletil (Virbac Laboratory, Carros-France). The present research was approved by the Animal Care and Use Committee (IACUC), Faculty of Science, Mahidol University. Animal Protocol number MUSC60-044-394. Central Animal Facility, Faculty of Science, Mahidol University has been international accredited by AAALAC. All experiments and methods used in the present study were performed in accordance with relevant regulations, and the ARRIVE (Animal Research: Reporting of In Vivo Experiments) guideline.

### Experimental design

#### Experiment 1: bone impairment in inadequate dietary calcium intake

To test whether inadequate dietary calcium intake in growing period caused impairment of bone growth in length and width, and whether bone impairment was more severe if calcium insufficiency lasted until young adulthood. Four-week-old female rats were randomly allocated into 2 groups (n = 16 each), i.e., calcium-replete diet (denoted as *S1,* a normal baseline group) and low calcium diet (denoted as *L*). As for *S1* group, rats fed with calcium-replete diet (0.55% w/w calcium) and oral gavage once daily with 20 IU/kg of 25(OH)D_3_ (Cat# C9756, Sigma) dissolved in food-grade soy bean oil. As for *L* group, rats fed with low calcium diet (0.15% w/w calcium) and oral gavage with soil bean oil to induce calcium insufficiency, thereafter, *S1* and *L* were euthanized at 2 weeks and 11 weeks after low calcium diet feeding (n = 8 each group, total of 32). Femora and tibiae were cleaned of adhering tissues, wrapped in normal saline-soaked gauze and kept frozen at –20 °C until analysis.

#### Experiment 2: calcium supplementation from CaCO_3_ and tuna bone

To test whether calcium supplement from tuna bone had higher intestinal calcium absorption efficiency than that of CaCO_3_, four-week female rats (n = 16) were fed with low calcium diet (0.15% w/w) for 2 weeks, after which the rats were switched to 2 formulae of calcium-replete diet (0.55% w/w) (n = 8 each). The extra 0.4% w/w calcium in the diet came from micronized purified CaCO_3_ (Konoshima chemical Co., Ltd., Osaka, Japan) (denoted as *S1*) or an equivalent amount of calcium from tuna bone (UniQ™, Thai Union Group Company PCL) (denoted as *S2*). After rats had been fed calcium sufficient diet for 4 weeks, intestinal fractional calcium absorption and urinary calcium excretion were investigated. All rats were subcutaneously injected with calcein (10 mg/kg, C0875, Sigma, St. Louis, MO) on 6- and 1-day before termination to examine dynamic bone formation. Tibiae were used for bone microstructural analysis by bone histomorphometry. Serum was kept frozen in − 80 °C for chemical analyses. Please note that, animal used in Experiment 2 was different set of animals used in Experiment 1.

#### Experiment 3: calcium supplementation from tuna bone with/without tuna head oil and 25(OH)D_3_

The objectives of this experiment were (i) to investigate the adaptive changes of intestinal calcium transport in response to low calcium diet with and without subsequent calcium supplementation, (ii) to test whether calcium supplementation was able to fully alleviate bone impairment caused by low calcium intake during young growing period, and (iii) to investigate possible additive effects of 25(OH)D_3_ supplement together with tuna calcium. Eight 4-week female rats were fed with either 0.55% w/w calcium-replete diet (*S1*) with daily 25(OH)D_3_ supplement (n = 8, a normal baseline) or 0.15% w/w low calcium diet (*L,* n = 32) for 2 weeks. Then, low calcium fed rats were randomly allocated to 4 groups (n = 8 each) that were fed the following diets (i) *L*, (ii) *L* mixed with tuna bone (denoted as *S2*), (iii) *L* mixed with tuna bone and oral gavage once daily with tuna head oil and 25(OH)D_3_ (denoted as *S3*) and (iv) *L* mixed with tuna bone and oral gavage once daily with 25(OH)D_3_ (Cat# C9756, Sigma) dissolved in food-grade soy bean oil (*S4*). As for *S3*, 25(OH)D_3_ was undetectable in refined tuna head oil because it was removed during the refining process. Therefore, we added 25(OH)D_3_ to the refined tuna head oil before giving to *S3* rats. 25(OH)D_3_ supplementation in *S3* and *S4* was 20 IU/kg BW. Rats were daily given soy bean oil with or without 25(OH)D_3_ or tuna head oil approximately 0.28–0.37 g/rat. Regarding to nutrition labeled, this small supplemental volume provided 2.47–3.27 cal/dose which was not expected to cause any metabolic or gastrointestinal disturbance. Intestinal fractional calcium absorption and urinary calcium excretion were measured before calcium supplementation and after 4- and 9-weeks of calcium supplementation. Body weight was recorded every 2 weeks. All rats were fed with designated diet for 11 weeks. At the end of experiment, rats were euthanized and femora were cleaned of adhering tissues, wrapped in normal saline-soaked gauze and kept frozen at − 20 °C until analysis. Tibiae were subjected to bone microstructural analysis by bone histomorphometry. Serum was kept frozen at − 80 °C for chemical analyses. Importantly, rats used in *S1* and *L* groups here were the same set of animals used in Experiment 1, while animals in groups *S2*, *S3* and *S4* were independent from previous Experiments (please see Supplemental Table 3 for summarize of animal used).

#### Experiment 4: additive effect of calcium supplementation from tuna bone with running exercise

To investigate whether combined running exercise and calcium supplementation provided a greater benefit to bone than either factor alone, forty 4-week female rats were fed low calcium diet for 2 weeks, thereafter, they were randomly allocated to 4 groups (n = 10 each), i.e., sedentary fed 0.15% w/w low calcium diet (*SL*), sedentary with 0.55% w/w calcium replete diet (*SB*), exercise fed 0.15% w/w low calcium diet (*EL*), and exercise with 0.55% w/w calcium replete diet (*EB*). Calcium replete diet used in Exp 4 was similar to diet in group *S2* in Exp 3. All rats were housed in cage-equipped with running wheel circumference of 144 cm and equipped with the electronic counter (model 80859, Lafayette Instrument Company, Lafayette, IN, USA). For the exercise groups, rats had free access to running wheel, whereas the sedentary groups were housed in cages with locked running wheels. The voluntary running program was modified from the method of Lapmanee et al.^61^. Briefly, on 1st week of exercise training, the running distance was monitored daily by reading from electronic counter display. Rats which ran less than 1 km/day was re-allocated to sedentary group with the same diet regimen. Rats performed exercise and were given calcium sufficient diet from tuna bone for 12 weeks during which running distance was recorded every 2 weeks. This wheel running protocol was considered a light-moderate intensity exercise with oxygen consumption of 74 ± 3.9 mL/min/kg^62^. Intestinal fractional calcium absorption and urinary calcium excretion were determined twice (i.e., in 6 weeks of exercise training and at the end of the experiment). Femora were wrapped in normal saline-soaked gauze and kept frozen at − 20 °C until analysis. Serum was kept frozen at − 80 °C until chemical analyses.

### Tuna bone powder and tuna head oil preparation

Tuna bone powder (UniQ™BONE, Thai Union Group, PCL.) was prepared as previously reported^16^. Briefly, after removing the remaining meat from tuna bone frames (*Katsuwonus pelamis*), backbones were crushed into small pieces and placed in 0.5% NaOH solution at 90–95 °C for 60 min, then rinsed with softened water until the pH of the water became 7.0. Small pieces of bone were further incubated with protease enzymes for 60 min, after which the mixture was heated to 90–95 °C and maintained at this range of temperature for 60 min to inactivate the enzymes. Then, pieces of bones were rinsed with water and dried until the moisture content was less than 5%. Thereafter, they were crushed in a fluidized bed opposed jet mills (model 100AFG; Hosokawa Alpine Aktiengesellschaft, Augsburg, Germany).

Tuna head oil was extracted with in-house protocol (Thai Union Group, PCL.). Briefly, after passing the standard quality control, fresh tuna heads were crushed into small pieces by coarse and fine grinding units, then crushed bones were mixed with hot water and agitated in the coagulation tank at 40 ± 2 °C for 45 min, then, grinded bones were pumped into oil separation unit (Three-phase Decanter unit), after which, oil phase at upper layer was then pumped into oil tank. This crude oil was filtered, centrifuged, cooled down until it was 25 ± 2 °C. It was then refined through in-house refining processes for removal of free fatty acids, impurities, odor and colour (UniQ™DHA, Thai Union Group, PCL.). Tuna head oil used in this study contained 33.90 ± 1.70% omega-3 (i.e., 26.24 ± 0.76% DHA and 5.34 ± 0.17% EPA).

### Determination of plasma free ionized calcium, total calcium, inorganic phosphate, serum bone turnover marker levels, 25(OH)D_3_ and 1,25(OH)_2_D_3_

For plasma ionized calcium analysis, blood was drawn with a commercial sterile heparinized syringe (model REF364314; BD, Diagnostics, Phymouth, UK). Plasma ionized calcium was determined by ion-selective electrode (model Stat Profile CCX; Nova Biomedical, Waltham, MA) under an anaerobic condition. For serum collection, whole blood was allowed to clot at room temperature and was centrifuged (model D-37520; Kendro Laboratory Products, Hanau, Germany) at 1500×*g* for 10 min at 4 °C. Clotted blood samples were used to determine total serum calcium and serum inorganic phosphate by *o*-cresolphthalein complexone method (model Dimension RxL analyzer, Dade Behring, Marburg, Germany). Bone turnover markers (i.e., P1NP and CTX-1), 25(OH)D_3_ and 1,25(OH)_2_D_3_ were measured by enzyme-linked immunosorbent assay kits, i.e., P1NP (catalog no. AC-33F1, Immunodiagnostic Systems, AZ, USA), CTX-1 (Cat# AC-06F1, Immunodiagnostic Systems, AZ, USA), 25(OH)D_3_ (Cat# KRR1971, DIAsource, Belgium) and 1,25(OH)_2_D_3_ (Cat# MBS160920, MyBiosource, USA).

### Bone histomorphometry

After cleaning of adhering tissues, tibiae were cut one-third in length, and immediately dehydrated in 70%, 95% and 100% vol/vol ethanol for 3 days at each concentration. Dehydrated tibiae were then embedded in methyl methacrylate resin in a posterior end down position, and incubated at 42 °C until the resin became fully polymerized. Undecalcified bone specimens were longitudinally cut into 7-µm-thick sections with a microtome equipped with tungsten carbide knife (model RM2265; Leica, Nussloch, Germany). Bone sections were then mounted on gelatin-coated microscope slides, deplastinated, dehydrated and stained with Goldner’s trichrome. Imaging analysis was performed under a light microscope using the computer-assisted Osteomeasure system (Osteometric Inc., Atlanta, GA, USA), operated with software version 4.1. The region of interest was secondary spongiosa (the trabecular part of proximal tibia at 2 mm distal to the epiphyseal plate), which was analyzed to obtain static parameters, i.e., trabecular bone volume normalized by tissue volume (BV/TV, %), trabecular thickness (Tb.Th, µm), trabecular separation (Tb.Sp, µm), trabecular number (Tb.N, mm^−1^), osteoblast surface normalized by bone surface (Ob.S/BS, %), osteoclast surface (Oc.S/BS, %) and active eroded surface (aES/BS, %). As for the dynamic parameters, undecalcified bone specimens were longitudinally cut into 12-µm-thick sections, deplastinated, dehydrated and were covered by standard cover slip. Unstained slides were analyzed for bone formation rate normalized by bone surface (BFR/BS, %) under fluorescence microscope (model eclipse Ni-U; Nikon).

### Bone mineral density and mineral content, and 3-dimension microstructural analysis by peripheral quantitative computed tomography and micro-computed tomography

Bone mineral density (BMD) and mineral content (BMC) were analyzed at femoral distal metaphyseal region and diaphyseal mid-shaft. Cortical microstructure (i.e., cortical thickness (Ct.Th), cortical area (Ct.A), cortical periosteal perimeter (Ct.Ps.Pm)), and endosteal perimeter (Ct.Es.Pm) was analyzed at femoral mid-shaft by peripheral Quantitative Computed Tomography (pQCT) in the research M mode (XCT Research SA+, Stratec Medizintechnik GmbH., Germany). Bones were scanned with 50 kV X-ray tube, current of 0.307 mA with voxel size of 0.1 mm^3^, and were analyzed by using XCT-5.50E software (Stratec, Medizintechnik GmbH., Germany). As for the metaphyseal region, measured parameters were averaged from three points of volume of interests (VOI) that were at 2, 2.5, and 3 mm away from the distal growth plate.

Three-dimensional trabecular metaphyseal microstructure was analyzed by ultra-high resolution micro-computed tomography (model UHR U-CT, Milabs, Utrecht, Netherlands). Measured parameters were bone volume normalized by tissue volume (BV/TV, %), trabecular thickness (Tb.Th, mm), and trabecular separation (Tb.Sp, mm) analyzed by Imalytics Preclinical software (version 13.0), and connectivity density (mm^−3^) and degree of anisotropy analyzed by using ImageJ program with BoneJ plugin.

### Mechanical property analyses

Femora were tested by 3-point bending technique (model 5943; Instron, Norwood, MA, USA). Femoral length was recorded before the mechanical test. Cross-head displacement rate was 2 mm min^−1^. Tests were conducted on the mid-diaphysis of the femora, which was placed on two supports 16 mm apart and with the femoral anterior margins facing downward toward the actuator. Recorded parameters were ultimate load, yield load, ultimate displacement, stiffness, Young’s modulus and stress and strain at maximum load. All mechanical parameters of each femur were automatically generated by Instron BlueHill Software (version 3.0, Nikon).

### Calcium determination in feces and urine by flame atomic absorption spectrophotometry

Rats were housed in individual metabolic cage (Techniplast, Venice, Italy) for 3 days to collect fecal pellets and urine. Fecal pellets were dried in an oven at 80 °C for 3 days and then ashed at 800 °C overnight in a muffle furnace (model 48000; Thermolyne, Dubuque, IA). Fecal dry and ash weight were recorded. Fecal ash was dissolved in acid solution and underwent microwave digestion at 220 °C, 1800 W for 35 min (Microwave acid digestion system, Ethos Up, Milestone Inc, CT). Feces and urine calcium contents were determined by flame atomic absorption spectrometry (PerkinElmer, MA, USA).

Fractional calcium absorption was calculated as followed

$${\text{Fractional}}\;{\text{calcium}}\;{\text{absorption}}\;(\% ) = \frac{{{\text{Calcium}}\;{\text{intake}}\;{\text{(g)}} - {\text{fecal}}\;{\text{calcium}}\;{\text{excretion}}\;{\text{(g)}}}}{{{\text{Calcium}}\;{\text{intake}}\;{\text{(g)}}}} \times 100$$

where, calcium intake is amount of calcium intake from diet over 3-day balance study, fecal calcium excretion is the amount of calcium presented in feces over 3-day balance study.

### Determination of intestinal calcium transporter protein expression by immunofluorescent

Duodenal segments were fixed overnight in 0.1 M phosphate-buffered saline (PBS) containing 4% paraformaldehyde. After being embedded in paraffin, the specimens were cut longitudinally into 4-µm sections which were later incubated at 95 °C for 20 min in sodium citrate buffer solution (pH 6.0). Non-specific bindings were blocked for 2 h by 4% bovine serum albumin, 5% normal goat serum, and 0.1% Tween-20 in PBS. Thereafter, sections were incubated at 4 °C overnight in moist chamber with rabbit monoclonal anti-PMCA1 (plasma membrane Ca^2+^ ATPase) at 1:500 dilution (Cat# ab190355, Abcam, RRID:AB_2893200). After being washed with 0.5% Tween-20 in PBS, sections were incubated for 1 h at room temperature in the dark with anti-rabbit Dylight 594 (Cat#DI-1594, Vector Laboratories) in blocking buffer. Sections were mounted with slow fade Diamond antifade mounting medium containing DAPI (Cat# S36964, Invitrogen) visualized under a fluorescent microscope (model eclipse Ni-U; Nikon). Fluorescent intensity was measured from 5 images per rat, 3–4 villi per image (total of 15–20 villi/rat), 5 rats/group by NIS-elements BR imaging software (version 4.0).

### Statistical analysis

Results are expressed as means ± standard errors (SE). All bar graphs showed individual data as scatter dots. The differences between two sets of data were analyzed by two-tailed unpaired Student’s *t* test. One-way analysis of variance with Tukey’s multiple comparisons test was used for multiple sets of independent data. The interaction of tuna bone calcium supplementation vs. running exercise was analyzed by two-way analysis of variance (two-way ANOVA), followed by Tukey’s multiple comparisons posthoc test. The level of significance for all statistical tests was *P* < 0.05. All data were analyzed by GraphPad Prism 9.4 for macOS (GraphPad Software, San Diego, CA).

## Supplementary Information

Supplementary Information.

## Data Availability

The datasets generated and/or analyzed during the current study are not publicly available due future patent filing, but are available from the corresponding author on reasonable request.
