# Supplementary material for: Running exercise with and without calcium supplementation from tuna bone reduced bone impairment caused by low calcium intake in young adult rats
Source: Sci Rep. 2023 Jun 13;13:9568. doi: 10.1038/s41598-023-36561-y (PMC10264423; doi:10.1038/s41598-023-36561-y)
Supplement: Supplementary file 1 — Supplementary Information. [file 41598_2023_36561_MOESM1_ESM.pdf]

Running exercise with and without calcium supplementation from tuna bone reduced bone impairment caused by low calcium intake in young adult rats

Panan Suntornsaratoon[1,2*], Thachakorn Thongklam[3], Thaweechai Saetae[4], Buapuengporn Kodmit[1,2], Sarawut Lapmanee[5], Suchinda Malaivijitnond[4,6], Narattaphol Charoenphandhu[1,2,7,8], Nateetip Krishnamra[1,2]

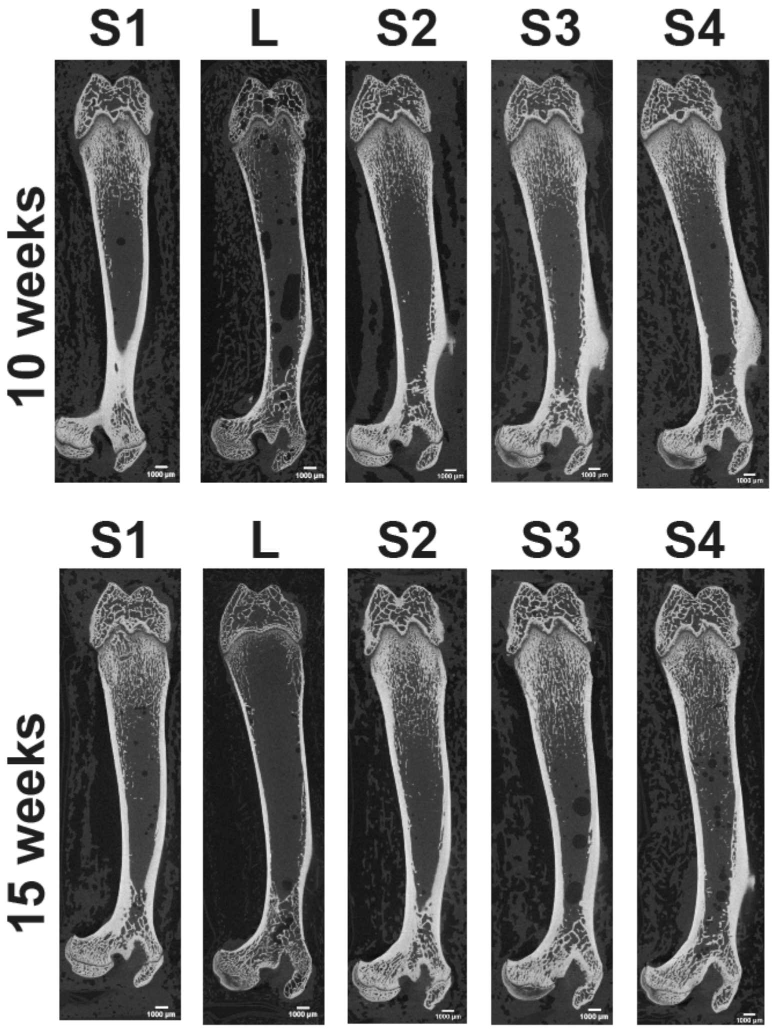

Supplemental Figure 1. Representative images of three-dimensional reconstruction of metaphyseal distal femur (longitudinal view) of 4 weeks calcium supplementation (upper panel) and 9 weeks calcium supplementation (lower panel) from tuna bone with or without 25(OH)D_3. Scale bars, 1 mm. Four-week-old female rats were randomly allocated into 2 groups, i.e., calcium-replete diet (denoted as *S1,* a normal baseline group) and low

calcium diet (denoted as *L*). 4-week female rats were fed with either 0.55% w/w calcium-replete diet (*S1*) with daily 25(OH)D_3 supplement (a normal baseline) or 0.15% w/w low calcium diet (*L*) for 2 weeks. Then, low calcium fed rats were randomly allocated to 4 groups that were fed the following diets *i*) *L*, *ii*) *L* mixed with tuna bone (denoted as *S2*), *iii*) *L* mixed with tuna bone and oral gavage once daily with tuna head oil and 25(OH)D_3 (denoted as *S3*) and *iv*) *L* mixed with tuna bone and oral gavage once daily with 25(OH)D_3 dissolved in food-grade soy bean oil (*S4*).

Preliminary study to fine effective treatment duration for calcium supplementation

In the beginning, we performed a preliminary study (figure below) in 4-week-old young growing female rats by investigating the alteration of femoral trabecular bone mineral density (BMD) by using an *in vivo* micro-computed tomography (Skyscan 1178). It showed that as soon as 2 weeks, low-calcium diet (0.15% w/w, *L*, n = 4) obviously caused a significant reduction in BMD as compared to age-matched rats fed calcium-replete diet (0.55% w/w, *S1*, n = 4). After bone loss was observed, calcium-deficient rats were randomly divided into 4 groups, i.e., *i*) remained on low-calcium diet (*L*), *ii*) switched to calcium-replete diet in which extra calcium came from tuna bone (*S2*), iii) as same as group *ii* with oral gavage of tuna head oil (*S3*), and *iv*) as same as group *ii* with oral gavage of 25(OH)D_3 (*S4*). All rats remained on designed diets and were investigated whether bone loss could be restored or fully regained to the same BMD level as in the age-matched normal rats (normal rats received normal calcium diet throughout the experimental period). In other words, we aimed to test the hypothesis whether bone loss induced by low calcium intake during childhood was permanent or not. It showed that calcium supplementation for 4 and 9 weeks was able to completely restored bone loss to age-matched normal control (animal were on age of 10 weeks and 15 weeks, respectively in supplemental figure 2). Therefore, we chose 9 weeks calcium supplementation for this study.

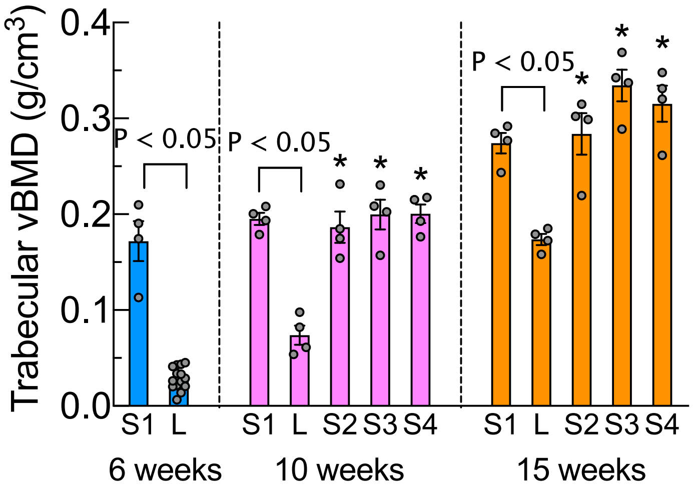

Supplemental Figure 2. Preliminary study to find effective treatment duration. * *P* < 0.05 compared with age-matched *L* group.

Supplemental table 1. Detail of calcium supplement diets.

Formulae	Detail	Total calcium in diet (%, w/w)	Source of extra calcium	Present in Figure or table
L	Low calcium diet	~ 0.15	–	Figures 2–6, Tables 1–3
S1	L mixed with 0.4% w/w extra calcium from $CaCO_3$	~ 0.55	$CaCO_3$	Figures 1–4, Table 1–2
S2	L mixed with 0.4% w/w extra calcium from tuna bone	~ 0.55	Tuna bone	Figures 1–6, Tables 2–3
S3	Similar to S2 and oral gavage once daily with tuna head oil and 25(OH)D_3	~ 0.55	Tuna bone	Figure 2–4, Table 2
S4	Similar to S2 and oral gavage once daily with 25(OH)D_3	~ 0.55	Tuna bone	Figure 2–4, Table 2

Supplemental table 2. Diet ingredients.

Contents	Diet formulae			Reference methods
	Low calcium diet (*L*)	Calcium supplement from $CaCO_3$ (S1)	Calcium supplement from tuna bone (S2)	
Calcium (% w/w)	0.15±0.01	0.55±0.05	0.54±0.08	AOAC (2012), 984.27
Vitamin D_3 (IU/kg)	800	810	830	AOAC (2012), 995.05
% Protein	25.4	13.0	12.4	AOAC (2012), 981.10
% Ash	4.6	4.8	5.2	AOAC (2012), 942.05
% Carbohydrate	59.6	73.5	73.3	Method of Analysis for Nutrition Labeling (1993) p.106
% Fat	4.7	2.6	2.7	AOAC (2012), 954.02
% Moisture	5.7	6.1	6.4	AOAC (2012), 930.15
Energy (kcal/100g)	382	369	367	Method of Analysis for Nutrition Labeling (1993) p.106

Supplemental table 3. Experimental groups.

Figures & Tables	Experimental groups				Remarks
	Experiments 1–3			Experiment 4	
	6 weeks	10 weeks	15 weeks	12&18 weeks	
Figure 1 and supp table 4	–	S1, S2	–	–	Rats used in Fig 1 were independent from all other figures and did not present in anywhere else.
Figure 2–4	–	–	S1, L, S2, S3, S4	–	Rats aged of 15 weeks were the same set of animals as used in Table 1 (only S1&L) and Table 2.
Figures 5&6 and Table 3	–	–	–	SL, SB, EL, EB	Rats used in these figures and table 3 were independent from all other figures.
Table 1	S1, L	–	S1, L	–	- Rats aged of 6 weeks were independent from all other figures and did not present in anywhere else.<br>- Rats age of 15 weeks were the same set of animals as used in Figure 2 (only S1&L).
Table 2	–	S1, L, S2, S3, S4	S1, L, S2, S3, S4	–	- Rats aged of 10 weeks were independent from all other figures.<br>- Rats aged of 15 weeks were the same set of animals as used in Figs 2–4 and table 1 (only S1&L).

Supplemental Table 4. Body weight, tibial length, bone mechanical properties, bone mineral density (BMD) and content (BMC), 3D microstructure, and blood chemistry of rat fed calcium supplement from tuna bone compare with standard $CaCO_3$. ### $P < 0.001$ compared with body weight of 6 weeks.

	**$CaCO_3$ (*S1*)**		**Tuna bone (*S2*)**		
	**Mean ± SEM**	***n***	**Mean ± SEM**	***n***	***P value***
Body weight (g)					
6 weeks	172.2±3.729	8	173.8±3.643	8	0.7591
10 weeks	234.4±2.595###	8	242.2±4.194###	8	0.1330
Bone length (mm)					
10 weeks	3.249±0.015	8	3.284±0.024	8	0.2445
Bone mechanical properties					
Maximal load (N)	102.4±2.322	8	106.2±1.231	8	0.1632
Ultimate displacement (μm)	1117±111.3	8	1173±67.96	8	0.6754
Stiffness (N/mm)	310.7±5.720	8	302.3±9.289	8	0.4525
Bone mineral density and content, and related parameters					
Metaphyseal distal femur					
TOT.BMD (g/cm^2)	0.7025±0.008	8	0.7188±0.013	8	0.3084
TOT.BMC (mg/mm)	13.03±0.319	8	14.33±0.615	8	0.0814
Tb.BMD (g/cm^2)	0.4185±0.007	8	0.4240±0.015	8	0.7442
Tb.BMC (mg/mm)	2.286±0.145	8	2.216±0.258	8	0.8163
Sub Ct.BMD (g/cm^2)	0.9025±0.008	8	0.8950±0.015	8	0.6711
Sub Ct.BMC (mg/mm)	10.18±0.329	8	11.67±0.728	8	0.0841
Ct.BMD (g/cm^2)	0.9506±0.007	8	0.9443±0.012	8	0.6526
Ct.BMC (mg/mm)	10.42±0.403	8	11.48±0.513	8	0.1276
Ct.Th (mm)	0.8005±0.033	8	0.8761±0.050	8	0.2322
Ct.Ps.Pm (mm)	16.23±0.123	8	16.70±0.209	8	0.0692
Ct.Es.Pm (mm)	11.20±0.195	8	11.20±0.299	8	0.9986
Diaphyseal midshaft femur					
Ct.BMD (g/cm^2)	1.246±0.005	8	1.254±0.006	8	0.2741
Ct.BMC (mg/mm)	6.290±0.105	8	6.410±0.183	8	0.5789
Ct.A (cm^2)	5.050±0.081	8	5.140±0.127	8	0.5611
Ct.Th (mm)	0.5134±0.009	8	0.5020±0.011	8	0.4508
Ct.Ps.Pm (mm)	11.46±0.081	8	11.82±0.169	8	0.0723
Ct.Es.Pm (mm)	8.233±0.108	8	8.668±0.184	8	0.0608
Blood chemistry					
25(OH)D_3 (ng/mL)	46.80±9.865	6	85.74±15.33	6	0.0584
1,25$(OH)_2D_3$ (nM)	1.276±0.091	4	1.948±0.260	7	0.0937
Total calcium (mM)	2.621±0.027	8	2.570±0.035	8	0.2712
Inorganic phosphate (mM)	2.546±0.036	8	2.248±0.096	8	**0.0096**

Supplemental Table 5. List of abbreviations of bone-related parameters

Abbreviations	Parameters
BFR/BS	Bone formation rate normalized with bone surface
BMC	Bone mineral content
BMD	Bone mineral density
BV/TV	Bone volume normalized with tissue volume
Conn.D	Connectivity density
Ct	Cortical region
Ct.Th	Cortical Thickness
Ct.A	Cortical area
DA	Degree of anisotropy
dL.S/BS	Double-labeling surface normalized with bone surface
Es.Pm	Endosteal perimeter
MAR	Mineral apposition rate
Ob.S/BS	Osteoblast surface normalized with bone surface
Oc.S/BS	Osteoclast surface normalized with bone surface
Ps.Pm	Periosteal perimeter
Sub.Ct	Subcortical and cortical region
Tb	Trabecular region
Tb.N	Trabecular number
Tb.Th	Trabecular thickness
Tb.Sp	Trabecular separation
TOT	Total tissue
